# Reaffirming a Positive Correlation Between Number of Vaccine Doses and Infant Mortality Rates: A Response to Critics

**DOI:** 10.7759/cureus.34566

**Published:** 2023-02-02

**Authors:** Gary S Goldman, Neil Z Miller

**Affiliations:** 1 Epidemiological Research, Independent Computer Scientist, Bogue Chitto, USA; 2 Medical Research, Institute of Medical and Scientific Inquiry, Santa Fe, USA

**Keywords:** non-specific effects, infant mortality rates, hepatitis b vaccine, child immunization, vaccines, vaccination rates, linear regression analysis, hdi- human development index, coefficient of determination, confounders

## Abstract

Introduction

In 2011, we published a study that found a counterintuitive, positive correlation, *r *= 0.70 (*p* < .0001), demonstrating that among the most highly developed nations (*n* = 30), those that require more vaccines for their infants tend to have higher infant mortality rates (IMRs). Critics of the paper recently claimed that this finding is due to "inappropriate data exclusion," i.e., the failure to analyze the "full dataset" of all 185 nations.

Objective

In the present study, we examine various claims postulated by these critics and the validity of their scientific methods, and we perform several investigations to assess the reliability of our original findings.

Methods

The critics select 185 nations and use linear regression to report a correlation between the number of vaccine doses and IMRs. They also perform multiple linear regression analyses of the Human Development Index (HDI) vs. IMR with additional predictors and investigate IMR vs. percentage vaccination rates for eight different vaccines. We perform odds ratio, sensitivity, and replication analyses.

Results

The critics' reanalysis combines 185 developed and Third World nations that have varying rates of vaccination and socioeconomic disparities. Despite the presence of inherent confounding variables, a small, statistically significant positive correlation of *r* = 0.16 (*p *< .03) is reported that corroborates the positive trend in our study. Multiple linear regression analyses report high correlations between IMR and HDI, but the number of vaccine doses as an additional predictor is not statistically significant. This finding is a likely consequence of known misclassification errors in HDI. Linear regression of IMR as a function of percentage vaccination rates reports statistically significant inverse correlations for 7 of 8 vaccines. However, several anomalies in the scatter plots of the data suggest that the chosen linear model is problematic.

Our odds ratio analysis conducted on the original dataset controlled for several variables. None of these variables lowered the correlation below 0.62, thus robustly confirming our findings. Our sensitivity analysis reported statistically significant positive correlations between the number of vaccine doses and IMR when we expanded our original analysis from the top 30 to the 46 nations with the best IMRs. Additionally, a replication of our original study using updated 2019 data corroborated the trend we found in our first paper (*r* = 0.45, *p =* .002).

Conclusions

A positive correlation between the number of vaccine doses and IMRs is detectable in the most highly developed nations but attenuated in the background noise of nations with heterogeneous socioeconomic variables that contribute to high rates of infant mortality, such as malnutrition, poverty, and substandard health care.

## Introduction

In 2011, we [1] observed that the United States required more vaccine doses for infants than any other nation, yet several nations had better (lower) infant mortality rates (IMRs). We conducted a study to explore a potential association between the number of vaccine doses that these 30 nations (the US and all nations with better IMRs) routinely give to their infants and their IMRs. Linear regression analysis yielded a coefficient of determination, *r*^2^ = 0.49 (*r* = 0.70; *p* < .0001). This positive correlation was counterintuitive: among the most highly developed nations, those requiring the most vaccine doses for their infants tended to have the highest IMRs.

Dr. E. Bailey, a professor at Brigham Young University (BYU), and several students associated with her Bioinformatics Capstone course recently read our study and found it "troublesome that this manuscript is in the top 5% of all research outputs." They reanalyzed our study to "correct past misinformation" and claimed that its findings were due to "inappropriate data exclusion," i.e., failure to analyze the "full dataset" of all 185 nations. The "Bailey reanalysis" was posted on the medRxiv preprint server [2] on September 10, 2021 (version 1), October 5, 2021 (version 3), and December 2, 2022 (version 4), which we refer to in this paper.

One stated rationale behind Bailey's reanalysis (and additional new investigations) is to reduce the impact of vaccine hesitancy, which "has intensified due to the rapid development and distribution of the COVID-19 vaccine." They also appear to be targeting our study for a potential retraction.

Our present study examines the various claims postulated by Nysetvold et al. and assesses the credibility of their methodology, analyses, reported results, and conclusions. Additionally, we provide odds ratio, sensitivity, and replication analyses, which reinforce and corroborate the methodology and findings presented in our original study.

## Materials and methods

The data discussed in this paper are based on our previous study [1] and the Bailey reanalysis [2]. Supplementary material is available at https://github.com/Miller-Goldman/Supplementary_Material.

Nysetvold et al. perform a linear regression analysis on 185 nations, do multiple linear regression analyses using IMR, HDI, and three predictors, and do linear regression analyses of IMR vs. percentage vaccination rate for each of eight different vaccines.

To investigate the robustness of our original study, we provide an odds ratio analysis with data divided at the median IMR and total vaccine doses, controlling for 11 variables. Additionally, we perform a sensitivity analysis by incrementally adding nations, one at a time, with successively lower-ranked IMRs than the US, to the linear regression analysis of IMR vs. a number of vaccine doses, until such time as the reported *r*-value is no longer statistically significant.

We also replicated our original study (which was based on 2009 data) using 2019 data. IMRs were amassed from the UNICEF Data Warehouse. Immunization schedules and the number of infant doses required by each nation were collected from the World Health Organization, the European Centre for Disease Prevention and Control, and national governments [3-5]. Once again, the dataset included the US, a nation that required the most vaccines for their infants, and all nations with better IMRs than the US. Three nations reporting fewer than five infant deaths (Andorra, Monaco, and San Marino) were omitted from the analysis due to IMR instability.

## Results

Comparison of our original investigation of IMR and number of vaccine doses with the Nysetvold et al. reanalysis 

**Figure 1:**
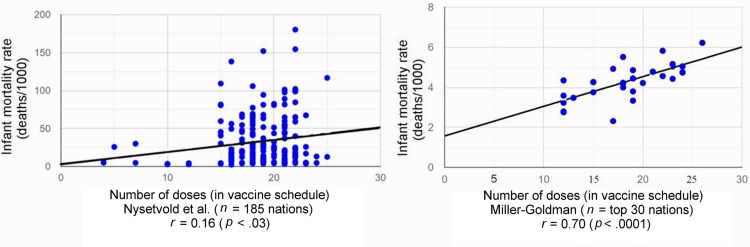
Scatter plot of infant mortality rates vs. number of vaccine doses with best-fit line and correlation coefficients Reported by Miller-Goldman [1] and Nysetvold et al. [2].

Linear regression analysis of IMR and the number of vaccine doses for each nation yields a statistically significant positive correlation of *r* = 0.70 (*p* < .0001) for nations with the top IMRs (*n* = 30) analyzed by Miller and Goldman, and *r* = 0.16 (*p* < .03) for the "full dataset" (*n* = 185) analyzed by Nysetvold et al. (Table 1, Figure 1).

**Table 1:** Data characteristics and linear regression analysis outcomes of original Miller-Goldman study and the reanalysis by Nysetvold et al. ^a^When the residuals are not normally distributed, the hypothesis is that they are not from a random dataset. The amount of residual error in the model is inconsistent across the full range of observed data. ^b^The model is sensitive to these outliers that represent poor quality data from Third World nations with the highest IMRs. This has the effect of changing the magnitude of the correlation coefficient and altering both the slope and y-intercept of the best-fit line. ^c^The linear regression analysis of Nysetvold et al. is irredeemably confounded due to varying vaccination rates and socioeconomic disparities between developed and Third World nations that have the effect of attenuating the significant positive correlation between IMR and number of vaccine doses that was found among nations with top-ranked IMRs.

Parameter	Linear regression analysis performed by	
	Miller and Goldman [1]	Nysetvold et al. [2]
Number of nations (*n*)	30	185
Appropriateness of data for linear regression analysis	Valid	Invalid (due to a trumpet or cone appearance of data points)
Number of unexplained nations missing from analysis	0	6 (Libya, Laos, Myanmar, South Africa, Venezuela, and Vietnam)
Number of nations excluded due to IMR instability	4 (Andorra, Liechtenstein, Monaco, and San Marino)	0 (Five nations with IMR instability were not excluded)
Range of IMRs	2.31 to 6.22	2.31 to 180.21
Best-fit equation	*y* = 1.6 + 0.15*x*	*y* = 2.7 + 1.6*x*
Correlation direction	Positive	Positive
Correlation coefficient (*r*)	0.70 (95% CI: 0.51 to 0.89)	0.16 (95% CI: 0.018 to 0.30)
p-value	< .0001	< .03
Coefficient of determination (*r*^2^)	0.49	0.026
Residual normality	Normal	Not normal^a^
Outliers	0	4 (Afghanistan, Angola, Liberia, and Sierra Leone)^b^
Vaccination rate of selected nations	Consistently >90%	Widely variable (<40% to >90%), resulting in confounding
Uniformity of socioeconomic factors of selected nations	Homogeneous	Heterogeneous
Robustness of results	Primarily reflects influence of vaccine doses (minimal influence of socioeconomic confounders)	Confounded by factors other than vaccine doses (i.e., varying vaccination rates and socioeconomic disparities)^c^

Additional investigations by Nysetvold et al. (not associated with our original study)

Association Between IMR and HDI

Two classifications of human development (high and very high) were combined to investigate an association between IMR and HDI using two subsets of 78 and 55 nations. The latter analysis, which included three covariates, reported *r*^2^ = 0.67, with none of the covariates reaching statistical significance. We replicated Bailey's investigation of IMR and HDI selecting only "very high" developed nations; the correlation declined to *r*^2^ = 0.39, about 42% less than the value they reported.

Association Between IMR and Percentage Vaccination Rate

**Figure 2:**
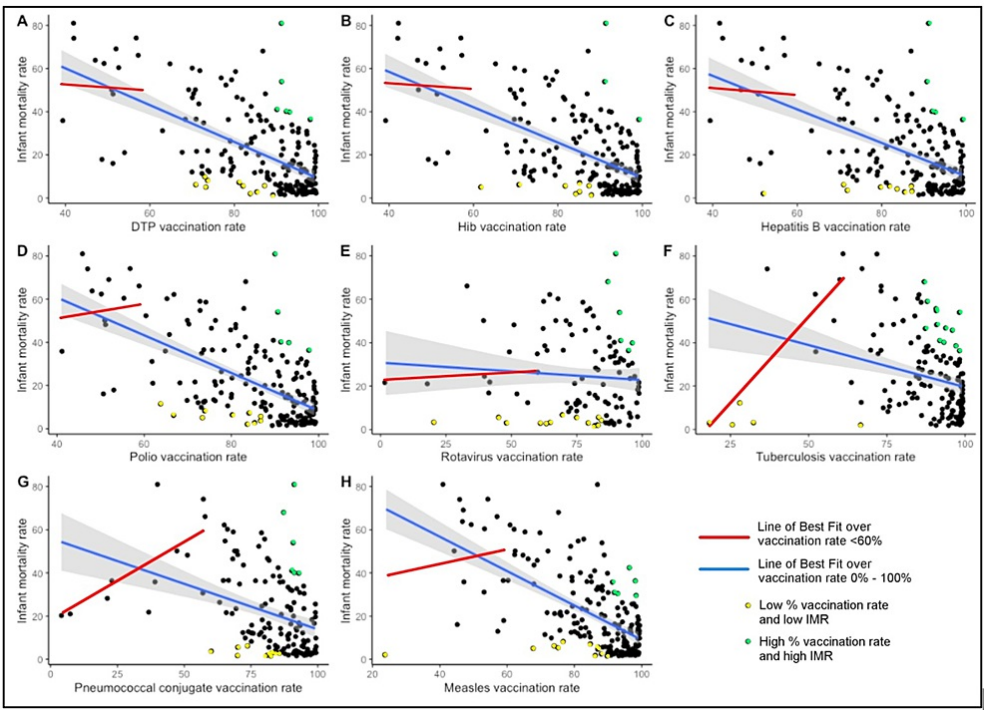
Line of best fit for vaccination rates <60% (red) and for all vaccination rates (blue), with colored data points showing that high vaccination rates are neither necessary nor sufficient to cause low infant mortality rate

The Bailey team's linear regression analyses (for the year 2019) of IMRs as a function of vaccination rates for each of eight different vaccines reports statistically significant inverse correlations for 7 of 8 vaccines. No correlation was found between IMR and percentage vaccination rate for the rotavirus vaccine (Figure 2).

Our three new investigations

Odds Ratio Analysis

A statistician, Dr. Walter Schumm, independently conducted an odds ratio investigation whereby nations in our study were divided at the median IMR and total vaccine doses, controlling for several confounding variables (including low birth weight, child poverty, and breast feeding). The correlation between each of the control variables and partial correlations are shown in Table 2. None of these factors lowered the original correlation below 0.62, thus robustly confirming our findings.

**Table 2:** Controls for relationship between number of vaccine doses and infant mortality rates in our original study (n = 30) IMR: infant mortality rate.

Control variable (number of nations providing data)	Correlation between control variable and vaccine doses	Correlation between control variable and IMR	Partial correlation between vaccine doses and IMR controlling for control variable(s)
Child poverty rate (28)	.41 (*p* < .03)	.46 (*p* < .02)	.68 (*p* < .001)
Low birth weight (28)	.26	.25	.72 (*p* < .001)
Pertussis vaccination rate (24)	−.56 (*p* < .005)	−.56 (*p* < .006)	.64 (*p* < .002)
Breast feeding ever % (22)	−.50 (*p* < .02)	−.37 (*p* < .10)	.73 (*p* < .001)
Teenage fertility rate (28)	.44 (*p* < .02)	.48 (*p* < .01)	.67 (*p* < .001)
Births out of wedlock (27)	−.13	−.21	.73 (*p* < .001)
Average age at first marriage (22)	−.46 (*p* < .04)	−.36 (*p* < .11)	.62 (*p* < .004)
No children when divorced % (21)	−.17	.12	.70 (*p* < .002)
Total fertility rate (27)	.11	.08	.74 (*p* < .001)
Pertussis incidence (24)	−.13	.05	.75 (*p* < .001)
Mother's index (29)	.13	.24	.74 (*p* < .001)

Sensitivity Analysis (Successive Analyses of Nations With Worse IMRs Than the US)

**Figure 3:**
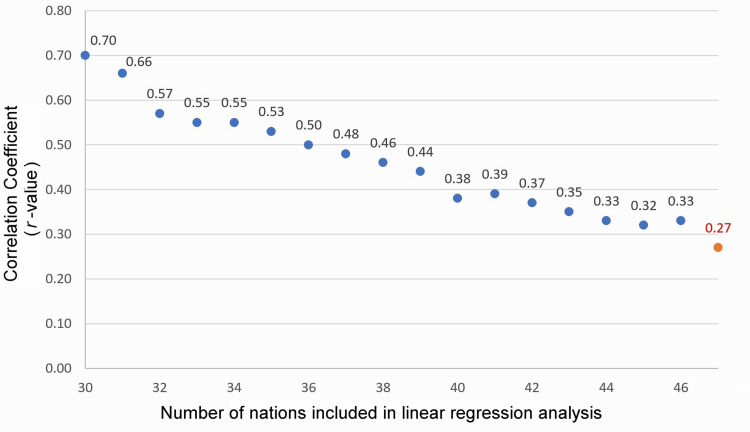
Correlation coefficients resulting from successive linear regression analyses of nations with incrementally worse infant mortality rates

A linear regression analysis was first performed on the top 30 nations using IMRs and vaccine doses as originally reported in our study. This produced the first data point shown in the top left of Figure 3, corresponding to a reported correlation coefficient of *r* = 0.70 (*p* < .0001). Next, using IMRs and vaccine doses derived from the same sources, linear regression analyses continued to be performed successively on each of the top 31, 32, 33,.... nations until the reported *r*-value was no longer statistically significant (at *n* = 47). The correlation coefficient (*r*) decreased from *r* = 0.70 (*p* < .0001) for *n* = 30 to *r* = 0.29 (*p* = .0504) for *n* = 47, as shown in Figure 3 and Table 3.

While our original study analyzed the top 30 nations with the US as the cutoff, an additional 16 nations (from Croatia #31 to Russia #46) could have been included in the linear regression of IMRs vs. the number of vaccine doses, and the findings would still have yielded a statistically significant positive correlation coefficient.

**Table 3:** Sensitivity analysis: successive linear regression analyses of nations with top infant mortality rates and the resultant r-values (2009 data) ^a^As nations with increasingly worse IMRs are added to the linear regression model, the correlation coefficients incrementally decrease (and *p*-values increase), likely due to worsening socioeconomic conditions and confounding. ^b^Not statistically significant.

Number of nations in regression (*n* = 30-47)	Cutoff nation	2009 IMRs	Doses	*r*-value^a^	*p*-value
30	United States	6.22	26	0.70	< .00002
31	Croatia	6.37	19	0.66	.00006
32	Belarus	6.43	16	0.57	.0006
33	Lithuania	6.47	19	0.55	.001
34	Serbia	6.75	19	0.52	.002
35	Poland	6.80	19	0.50	.002
36	Slovakia	6.84	19	0.48	.003
37	Estonia	7.32	19	0.46	.004
38	Chile	7.71	22	0.48	.002
39	Hungary	7.86	16	0.41	.009
40	Costa Rica	8.77	22	0.43	.005
41	Latvia	8.77	19	0.41	.008
42	Kuwait	8.97	19	0.39	.011
43	Ukraine	8.98	19	0.37	.014
44	Bosnia and Herzegovina	9.10	19	0.36	.016
45	Cyprus	9.70	21	0.37	.012
46	Russia	10.56	16	0.30	.040
47	Uruguay	11.32	19	0.29	.0504^b^

Replication Study Using 2019 Data

**Figure 4:**
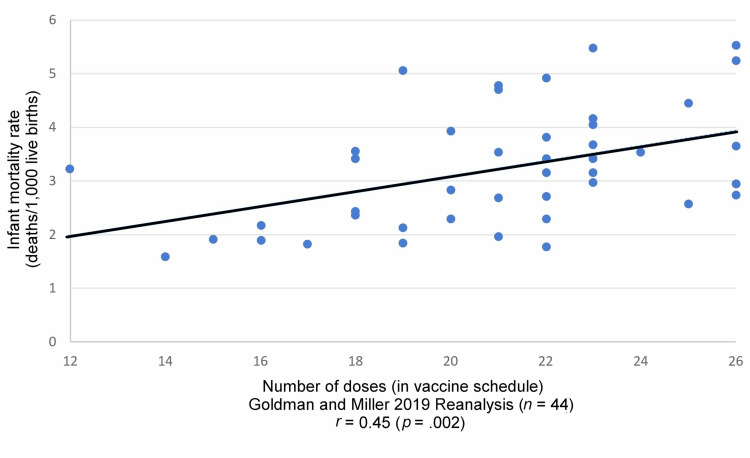
Linear regression analysis of infant mortality rates and infant vaccine doses per nation reported by Goldman and Miller, 2019 data

Using 2019 data for the top 44 nations (the US and all nations with better IMRs), a linear regression analysis was performed to replicate our original study. We found *r* = 0.45 (*p* = .002); coefficient of determination, *r*^2^ = 0.20, corroborating the trend reported in our initial study (Figure 4 and Table 4). When the linear regression analysis was limited to the top 20 nations (range of IMRs = 1.59 to 2.95), the correlation coefficient increased (*r* = 0.73; *p* < .0003), revealing a strong direct relationship between IMRs and the number of vaccine doses.

**Table 4:** Infant mortality rates and number of vaccine doses per nation used in the 2019 replication of our original study

Nations	2019 IMRs	Doses	Nations	2019 IMRs	Doses
Iceland	1.59	14	Germany	3.16	22
Estonia	1.77	22	Denmark	3.24	12
Slovenia	1.82	17	France	3.41	22
Japan	1.85	19	Belgium	3.42	23
Norway	1.88	16	Hungary	3.42	18
Finland	1.91	15	Latvia	3.53	24
Singapore	1.97	21	Netherlands	3.53	21
Montenegro	2.13	19	Switzerland	3.55	18
Sweden	2.18	16	Greece	3.65	26
Cyprus	2.29	20	United Kingdom	3.68	23
Luxembourg	2.30	22	Poland	3.81	22
Belarus	2.36	18	Croatia	3.94	20
Czechia	2.43	18	New Zealand	4.05	23
Italy	2.58	25	Cuba	4.17	23
South Korea	2.68	21	Canada	4.44	25
Spain	2.71	22	Russia	4.70	21
Ireland	2.73	26	Slovakia	4.77	21
Portugal	2.84	20	Serbia	4.93	22
Austria	2.94	26	Bosnia and Herzegovina	5.06	19
Lithuania	2.95	26	Qatar	5.25	26
Israel	2.96	23	Bulgaria	5.49	23
Australia	3.16	23	United States	5.52	26

## Discussion

Biases in the Bailey reanalysis

Bailey and her coauthors erroneously claim that the main finding in our study is due to "inappropriate data exclusion," i.e., failure to analyze the "full dataset" of all 185 nations. It is essential to understand the biases in their "reanalysis," which is in fact a new analysis using completely different selection criteria.

#1: The Bailey team indiscriminately combined highly developed and Third World nations without regard to confounding arising from heterogeneous data. Clusters of heterogeneous nations (due to variable socioeconomic factors) report widely differing IMRs yet prescribe the same number of vaccine doses for their infants. For example, Angola (with an IMR of 180.21) and Belgium (with an IMR of 4.44) both require 22 vaccine doses.

#2: The Bailey reanalysis contains highly variable vaccination coverage rates but was not adjusted for confounding. For example, WHO/UNICEF (the resource used by the Bailey team to calculate vaccine doses) indicated that Chad had a national vaccination coverage rate that ranged from just 10% for three doses of the hepatitis B vaccine to 40% for the tuberculosis (BCG) vaccine. This was not a factor in our original study.

#3: The dataset of 185 nations used in the Bailey team's linear regression analysis contains outliers and produces residuals that are not normally distributed (Table 1) based on a Shapiro-Francia test of normality (W' = 0.0524, *p* < .0001), exhibiting an asymmetrical (right/positive) skewness. Outliers and nations with substandard living conditions should have been removed from the analysis, resulting in less IMR variability and retaining for analysis only those nations with high vaccination rates and homogeneity of socioeconomic factors.

#4: The Bailey reanalysis contains heteroscedastic data. Due to the dissimilar values of IMR over the range of the number of vaccine doses, Bailey's data do not have a constant variance (homoscedasticity). This leads to less precise coefficient estimates, and statistical tests of significance are invalidated.

#5: The Bailey team's random sampling and "distribution of regression results" generated spurious results. According to them, our main finding of *r*^2^ = 0.49 is "statistically improbable." This analysis [2] (Figure 2) consisted of 50,000 iterations of randomly selecting 30 nations from among all 185 nations. This analysis is flawed because it mixed nations displaying heterogeneity of socioeconomic factors without regard to covariates.

#6: Several poorly developed and Third World nations in the Bailey reanalysis had a subnational distribution of specific vaccines (as indicated in the WHO/UNICEF summary of immunization schedules). Since the Bailey team chose not to count these vaccines in their totals, (1) it makes their calculation of vaccine doses unreliable, and (2) their vaccine totals are underestimated because some unknown percentage of the population received them. This type of confounding is similar to the Bailey team's previous error in combining nations with different vaccination coverage rates.

Part of the problem is that the UNICEF data provided for each nation's immunization schedule is not entirely accurate when doses calculated using this source are compared to doses actually administered. For example, regarding Indonesia, UNICEF reports that DTP and Hep B have subnational distribution (except for the birth dose of Hep B), so the Bailey team omitted these vaccines from their tally (as per their own criteria) and counted just seven doses (one BCG, one HepB, four oral polio, and one measles). Yet, all nations require diphtheria, tetanus, and pertussis (DTwP or DTaP) vaccines for their infants, and UNICEF reported that 77% and 78% of the Indonesian infant population received three doses of DTP and Hep B, respectively. Thus, these vaccines were administered to most Indonesian infants, and the true number of vaccine doses is 18 (one BCG, three diphtheria, three tetanus, three pertussis, three HepB, four oral polio, and one measles).

#7: Six nations are excluded from the "full" dataset of 191 nations without explanation.

#8: Five nations that should have been excluded from the dataset (due to IMR instability as a result of very few reported infant deaths) are included. Since each nation's observed IMR is considered an estimate of the true underlying mortality rate, this estimate is subject to chance variation. If a nation has very few births, the observed (i.e., reported) IMR may be very different from the true rate. Thus, it is a standard statistical convention for the CDC and other health agencies to discard such values that contribute to IMR instability. Prominent biostatistician and epidemiologist, Joel C. Kleinman, PhD, who served in the Office of Analysis and Epidemiology, National Center for Health Statistics, explains:

"A useful rule is that any rate based on fewer than 20 cases in the numerator [of the infant mortality rate] will have a 95% confidence interval, which is about as wide as the rate itself (that is, from 0.5r to 1.5r). In general, this means all that can confidently be said about an area (or country) with 20 deaths out of, say 1000 live births, is that the true rate is within 20 ± 10 per 1,000. Clearly, this is not precise information" [6].

Preprints of the Bailey reanalysis criticize us for excluding four such nations, despite this exclusion being an established epidemiological convention. (When these four nations are included in our analysis, it produces a negligible effect on the overall reported results.)

#9: The number of vaccine doses for some nations is substantially miscounted. For example, seven infant vaccine doses are reported for Australia when the true value is 22 (as indicated by WHO/UNICEF) or 24 (as indicated by the immunization schedule published by the Australian government). Incorrectly calculated vaccine doses, whether due to the exclusion of subnationally distributed vaccines or a manual miscount, are problematic.

We conclude that Bailey's reanalysis of the "full dataset" of 185 nations generated spurious results due to flawed selection criteria and confounded data. Despite confounding, Bailey's reanalysis of the "full dataset" corroborated the positive trend of the correlation we reported.

Our linear regression analysis (*n* = 30) and the "reanalysis" by Nysetvold et al. [2] (*n* = 185) both report a statistically significant positive correlation coefficient (Table 1 and Figure 1). As expected, the correlation coefficient reported by Nysetvold et al. is small, likely due to the numerous confounders they included in their dataset.

Our selection criteria and additional confirmatory analyses 

Our study had selection criteria that consisted of nations with high vaccination rates (mainly exceeding 90%) and homogeneity of socioeconomic factors that minimized confounders (Supplementary Table S1). We had no prior knowledge as to whether a relationship between IMR and the number of vaccine doses existed. According to Lesko et al., "When first exploring the existence of a causal effect, epidemiologists may purposefully undertake non-random sample selection to increase statistical efficiency, match or restrict important confounders, or allow estimation of subgroup effects." [7] Subsequently, odds ratio, sensitivity, and replication analyses were conducted, all of which corroborated the direction of the trend reported in our original study.

A Sensitivity Analysis Confirms Robustness of Our Study Over Range of 30 to 46 Nations

Our original study analyzed the top 30 nations with the US as the cutoff, yet an additional 16 nations could have been included in the linear regression of IMRs vs. the number of vaccine doses, and the findings would still have yielded a statistically significant positive correlation coefficient. As nations with higher IMRs are successively analyzed, the correlation coefficients incrementally decrease (Figure 3 and Table 3). At *n* = 47 and higher, corresponding to nations with an IMR >10.6 deaths/1,000 live births, the best-fit line F-statistic is no longer statistically significant. This observed trend is as expected because when nations with increasingly worse IMRs are selected for analysis, the model incrementally shifts from a homogeneous to a heterogeneous dataset.

Effect of Analyzing Additional Nations on the Slope of the Best-Fit Line

In our study of the top 30 nations in 2009, IMR increased by a factor of 0.15 deaths/1,000 live births for each vaccine dose added to the immunization schedule. This increase remains consistent, with the slope ranging from 0.14 to 0.20 deaths/1000 live births for each successive nation from the top 30 to the top 46 added to the analysis. In contrast, the slope of the best-fit line in Bailey's reanalysis of 185 nations is 1.6 deaths/1,000 live births. Thus, the IMR increases by a factor of 1.6 deaths/1,000 live births for each vaccine dose added to the immunization schedule, approximately 10-fold higher than that of the top 30 to 46 ranked nations.

In nations with high infant mortality, some vaccines (e.g., IPV and DTP) appear to have an inverse safety profile when administered out of sequence [8], a hypothesis that might explain the larger slope when 185 nations are analyzed. Aaby et al. [9] discovered that vaccines have non-specific effects (NSEs) that can increase or decrease mortality from infectious diseases not targeted by the vaccine. In nations with high mortality, 35 studies were analyzed to verify whether DTP increases mortality. All studies without survival bias confirmed that DTP-vaccinated children had significantly higher mortality than DTP-unvaccinated children. Mogensen et al. [10] investigated NSEs as well and found that all-cause infant mortality in Guinea-Bissau after three months of age doubled after DTP and oral polio vaccines were introduced (hazard ratio, HR = 2.12). Infant survival was significantly worse in three- to five-month-old infants who received DTP only (without an oral polio vaccine) compared with not-yet-DTP-vaccinated children (HR = 10.0). Differences in background factors did not explain the effect.

Bailey's supplemental analyses

The Bailey Team Violated Their Own Restriction Against Data Exclusion by Selecting Subsets of Nations to Analyze

The Bailey team considers data from 83 "high" and "very high" developed nations, as categorized by the Human Development Index (HDI) [11]. A second multiple linear regression analysis includes two additional predictors: a GINI index to account for income inequality and an HAQ index to account for healthcare access and quality. After excluding 28 nations due to missing covariate data, 55 of the original 185 nations were available for analysis. (Over 70% of the full dataset was excluded.) The Bailey team reported that three of the covariates (i.e., GINI, HAQ, and vaccine doses) did not achieve statistical significance. However, a reported negative correlation suggests that as vaccine doses increase, IMR decreases, but this finding is likely specious.

First, Wolff et al. [12] note that inherent to HDI are "changes in formula that can lead to severe misclassification in the categorization of low, medium, high, or very high human development countries." According to these economists, "HDI cut-off values seem arbitrary," and they quantify that 11%, 21%, and 34% of countries are misclassified due to three sources of error: (1) data updates, (2) income revisions, and (3) thresholds to classify a country's development status, respectively. Additionally, since HDI is an aggregate of educational levels, income per capita, and life expectancy, it is not unexpected that high inverse correlations, accounting for 85-92% of the variation in IMR as a function of HDI, have been reported in studies as early as 1997 [13], although causes for the high inverse correlations cannot be determined [14]. Thus, the use of HDI has inherent limitations: it is unable to discern specific clues to improving IMR and may conceal important disparities in medical or healthcare practices that underlie IMR trends.

Second, research by Ruiz et al. indicates that the use of the inequality-adjusted human development index (IHDI) is a more reliable predictor of IMR than the use of HDI [15]. Ghislandi et al. [16] are also critical of the accuracy and meaningfulness of HDI data and created an alternative index, the Human Life Indicator (HLI), to address HDI shortcomings. While Denmark was recently ranked 5th in the world by HDI, it fell to 27th place with HLI; the US was recently ranked 10th by HDI while HLI ranked it 32nd.

The Bailey Team’s Investigation of IMR vs. Percentage Vaccination Rates Contains Several Anomalies

Most of the graphs (Figure 2) of IMR vs. percentage vaccination rate for a given vaccine are better represented using a two-segment, piecewise linear model due to widely differing slopes of the best-fit lines through data below and above a 60% vaccination rate breakpoint [17]. The cluster of data points corresponding to vaccination rates >90% has a wide range in IMRs (heteroscedasticity), which contributes to a problematic linear regression model.

Numerous data points in all the graphs indicate that some nations with low vaccination rates have low IMRs, while other nations with high vaccination rates have high IMRs. Thus, high vaccination rates are neither necessary nor sufficient to cause low IMR. Scientists would likely want to explore why those nations appear to contradict the prevailing medical presumption that high vaccination rates yield healthier children (e.g., lower IMRs).

This investigation also disregards the effect of combining vaccines and the potential for additive or synergistic toxicity, which we considered by comparing vaccine schedules for each country. Nysetvold et al. dispute our claim that synergistic toxicity may occur when multiple vaccines are administered concurrently, citing four highly selective articles [2]. However, a CDC report on mixed exposures to chemical substances and other stressors, including prescribed pharmaceuticals, found that they may produce "increased or unexpected deleterious health effects." In addition, "exposures to mixed stressors can produce health consequences that are additive, synergistic, antagonistic, or can potentiate the response expected from individual component exposures" [18]. Studies have also recognized the potential for detrimental interactive effects when multiple vaccines are administered concurrently or in close succession [19,20].

Further, one cannot perform a reliable analysis of polio without separating live and inactivated vaccines, which have opposite effects on infant mortality [8,21]. Numerous studies have convincingly shown that DTP significantly increases all-cause infant mortality in Africa [8-10], a finding that contradicts the inverse correlation reported by the Bailey team, and the measles vaccine is not administered to infants in many nations; it is given after the outcome period when IMR is measured. These examples call into question the reliability of the Bailey team’s entire investigation of IMR vs. percentage vaccination rates.

The Bailey Team Misinterprets a Longitudinal Increase in Hepatitis B Vaccination Rates With Lowering IMR

The Bailey team reports that from 1996 to 2019 (a 23-year period), hepatitis B vaccination in the US was "consistently high," and the IMR dropped from 8.5% to 6% in males and from 7% to 5% in females. However, hepatitis B was never a high risk for infants. In 1996, a single infant death out of 2.9 million births was attributed to this disease [22], so the vaccine could never realistically be expected to contribute to a lowering of the IMR, and from 1960 to 1981 (a 21-year period before the hepatitis B vaccine was introduced), the IMR declined by 53%, a much steeper decline than after the vaccine was introduced [23]. The authors acknowledge there are "potential confounding factors," but simply adding this statement is insufficient to justify including this "analysis" in their manuscript.

Additional concerns

Supplementary Table S2 contains summaries of additional concerns associated with the Nysetvold et al. [2] paper, including (1) inappropriate and libelous statements, (2) misrepresented funding issues, and (3) technical inaccuracies.

Biological plausibility for an association between infant vaccines and sudden infant death

There is credible evidence that a subset of infants may be at increased risk of sudden infant death shortly after being vaccinated. In Japan, from 1970 through 1974, there were 37 documented sudden infant deaths following pertussis vaccinations, inciting parents and doctors to refuse the vaccine. In 1975, Japanese authorities reacted to these events by increasing the age of vaccination from three months to two years [24]. According to Cherry et al. [25], "The category 'sudden death' is instructive in that it disappeared following both whole-cell and acellular vaccines when immunization was delayed until a child was 24 months of age."

In 1987, Walker et al. [26] wrote that "the major finding of the present study" was a statistically significant increased risk of sudden infant death syndrome (SIDS) in the early post-vaccination period. Infants died at a rate more than seven times greater than expected in the period zero to three days following DTP vaccination when compared to the period beginning 30 days post-vaccination (RR = 7.3, 95 % CI 1.7-31).

A recent study published in Toxicology Reports [27] analyzed an association between pediatric vaccines and sudden infant death. Of the 2605 infant deaths reported to the Vaccine Adverse Event Reporting System (VAERS) from 1990 through 2019, 58% clustered within three days post-vaccination, and 78% occurred within seven days post-vaccination, confirming that infant deaths tend to occur in temporal proximity to vaccine administration. The excess of deaths during these early post-vaccination periods was statistically significant (*p* < 0.00001).

On July 10, 2017, the US Court of Federal Claims [28] issued a decision with regard to a claim filed with the Vaccine Injury Compensation Program (VICP). A male infant, J.B., received seven vaccines at his four-month well-baby visit. On the following day, he died. The medical examiner stated that the cause of death was SIDS. His parents filed a petition under the VICP. Petitioners allege that as a result of receiving vaccines for diphtheria, tetanus, acellular pertussis, polio, Hib, pneumococcal, and rotavirus, J.B. passed away from SIDS.

After listening to expert testimony by Dr. Douglas C. Miller, a neuropathologist, Special Master Thomas L. Gowen concluded that the petitioners "have demonstrated by a preponderance of the evidence that the vaccines can and likely did play a critical role in this child's death." Miller explained that when an infant receives one or more vaccines concurrently, as J.B. did, it evokes the production of cytokines. Physiological studies have shown that these can produce fever and inhibit the activity of 5-HT neurons in the medulla, causing prolonged apneas and interfering with auto-resuscitation. Dr. Miller noted that J.B. was a "healthy infant…developing normally." He was "immunologically normal." After receiving vaccines, cytokines circulated into the central nervous system and interacted with the hypothalamus to provoke fever and act in the brainstem," which was already deficient in serotoninergic drive for respiratory effort, leading to an apneic episode from which he did not recover, i.e., SIDS" [28].

Strengths and limitations

We conducted several analyses that corroborated the trend reported in our original study (Supplementary Table S3 summarizes these analyses). Our analyses are ecological comparisons that provide insight into the relationship between exposure and outcome at a population level, and even though most of the nations had 90-99% national vaccination coverage rates, we do not know whether it is the vaccinated or unvaccinated infants who are dying at higher rates. Even so, there is plausible biological and causal evidence that the observed correlation between IMRs and the number of vaccine doses routinely given to infants might be causal. Vaccines can evoke the production of cytokines, and physiological studies have shown that these can produce fever and inhibit the activity of 5-HT neurons in the medulla, causing prolonged apneas and interfering with auto-resuscitation [28]. Additional detailed infant autopsy studies may elucidate vaccination mechanisms that might be involved in or contribute to the mortality currently attributed to SIDS.

The source material for immunization schedules is occasionally inconsistent. For example, the WHO/UNICEF resource used to calculate vaccine doses appears to be less precise than immunization schedules provided by national governments (presumed to be the most reliable). These minor discrepancies could slightly increase or decrease the reported study correlation.

We calculated the total number of vaccine doses recommended for children but did not differentiate between the substances, or quantities of those substances, in each dose. Common vaccine substances include antigens (attenuated viruses, bacteria, and toxoids), preservatives (thimerosal, benzethonium chloride, 2-phenoxyethanol, and phenol), and adjuvants (aluminum salts).

Vaccines are designed to protect against specific diseases but may have NSEs that can increase or decrease mortality from infectious diseases not targeted by the vaccine. The most recent vaccine given (live vs. non-live) seems to exert the strongest beneficial or detrimental NSE [29,30]. These factors were not considered in any of the analyses performed either by us or Nysetvold et al. Global health officials do not test the sequence of recommended vaccines or their NSEs to confirm they provide the intended effects on child survival. More studies on this topic are recommended to determine the full impact of vaccinations on all-cause mortality.

## Conclusions

There is a positive correlation between infant vaccines and infant mortality rates. This relationship is most pronounced in analyses of the most highly developed homogenous nations but is attenuated in background noise in analyses of nations with heterogeneous socioeconomic variables. Health authorities in all nations have an obligation to determine whether their immunization schedules are achieving desired goals. More investigations into the health outcomes of vaccinated vs. unvaccinated populations and the effect of vaccinations on all-cause mortality are imperative.
